# Pharmacy robberies and occupational safety risks: A national cross-sectional study of community pharmacies

**DOI:** 10.1016/j.rcsop.2026.100721

**Published:** 2026-02-15

**Authors:** Mohanad Odeh, Dana M. Odeh, Enas Alkhader, Duaa Alzyoud

**Affiliations:** a Department of Clinical Pharmacy and Pharmacy Practice, Faculty of Pharmaceutical Sciences, The Hashemite University, P.O. Box 330127, Zarqa 13133, Jordan; b Department of Pharmacy, Faculty of Pharmacy, Jadara University, P.O. Box 733, Irbid 21110, Jordan; c Pharmacy School, Middle East University, Amman, Jordan

**Keywords:** Pharmacies, Robbery, Violence/prevention & control, Occupational risks, Risk factors

## Abstract

**Background:**

Community pharmacies face substantial exposure to workplace violence. However, pharmacy robberies remain critically under-studied. No robust investigation has examined robbery risk in community pharmacies in Jordan or the broader Middle East region, representing a significant research gap.

**Aim:**

To quantify robbery exposure in community pharmacies, identify perceived pharmacy-level risk factors for robbery, and compare risk perceptions between pharmacists with and without robbery exposure.

**Methods:**

Analytical cross-sectional survey. A self-administered questionnaire with demonstrated content validity (CVR ≥ 0.74) and internal consistency (Cronbach's α > 0.70) was used. Data from 975 respondents were analyzed using chi-square tests with Cramer's V and multivariable logistic regression; ethical approval was obtained from the institutional review board.

**Results:**

Robbery exposure was reported by 4.3% of respondents and was significantly more frequent among men and pharmacists aged >30 years (*p* < 0.01). The most frequently endorsed pharmacy-level robbery risk factors were late opening (81.2%), single staffing (77.3%), dispensing narcotics (77.2%) and absence of surveillance cameras (72.6%). In adjusted analyses, agreement that robbery risk depends primarily on offender-related personal factors (OR 6.28, 95% CI 2.48–15.90) and prior experience with aggressive customers (OR 3.11, 95% CI 1.25–7.76) emerged as the strongest independent correlates of robbery exposure. Encounters with customers perceived as dangerous (72.1%) or aggressive (68.0%) were also common.

**Conclusion:**

Robberies, alongside widespread dangerous and aggressive customer behavior, pose meaningful risks in community pharmacies. Differences between pharmacists exposed and not exposed to robbery in how they evaluated pharmacy-level robbery risk factors, together with the strong influence of offender- and behavior-related factors on robbery exposure underscoring the need for evidence-informed security policies, minimum staffing standards, and training to recognize and manage high-risk customer interactions.

## 1 Introduction

Pharmacy practice in retail community settings is increasingly recognized as a site of occupational violence1., 2., 3 and workplace aggression, with pharmacists exposed to both external aggression and criminal threats, including robbery and burglary,^1^,^2^.4., 5., 6 These risks underscore the precarious position of pharmacists within the broader healthcare system In addition to these external threats, pharmacies also contend with underreported internal vulnerabilities, such as drug diversion – redirection of medicines from legal supply chains to unauthorized or illegal use - and patient initiated behaviors that compromise safety and integrity,^1^,^2^.4., 5., 6, 7.

Several studies have documented high rates of verbal abuse, physical aggression, and other forms of workplace violence directed towards community pharmacy staff,1., 2., 3,^5^.^8^ Recent incidents in the United Kingdom further illustrate the risks faced by pharmacy staff.9., 10., 11., 12., 13. Reports indicate a continuing rise in pharmacy-related violence, with tangible consequences for both staff and operations. For instance, in 2025, nearly nine out of ten pharmacies in Ireland reported experiencing criminal incidents, with a substantial proportion subjected to repeated offenses.^9^ In Scotland, an incident was reported in which a man generated significant “fear or alarm” by shouting and swearing at pharmacy staff after being informed of a prescription delay.^13^ Evidence from Denmark further illustrates that patient behavior can materially influence safety risks in community pharmacies.^14^ In Saudi Arabia, pharmacists report substantial workplace violence, with nearly 40% indicating exposure to verbal abuse alone.^2^ In addition, they frequently encounter inappropriate requests, including customer demands for medicines that do not meet clinical or regulatory criteria.^15^ In Jordan almost 25% of pharmacists have encountered suspected illegal requests from consumers, this aligns with broader evidence of illicit medicine-seeking behavior in Jordan, where almost one-third of pharmacists reported receiving customer requests to obtain doping agents.^16^

Within this broader pattern of workplace violence, pharmacy robbery emerges as a distinct, pharmacy-specific security problem that requires focused investigation and targeted efforts,^1^.^6^ A recent study from South-East Europe (Croatia, Serbia, Bosnia and Herzegovina, and Montenegro) reported that 26% of community pharmacy staff had experienced at least one robbery during their career, and almost one-third of these (30%) had been exposed to robbery on more than one occasion.^1^ To address the critical issue of pharmacy robberies and advocate for the addition of a lecture on pharmacy robberies to pharmacy curricula, McFarland and Devine conducted a comprehensive analysis of this phenomenon. Their investigation underscored the scale of robbery incidents; an internet search for “pharmacy robberies 2022 and 2023” returned over 2000 news reports on investigations and arrests involving armed pharmacy robberies in the United States and Canada.^6^ Complementing this, more than a quarter of pharmacy students reported their workplace had been robbed, which supports a longitudinal data reveal that between 2010 and 2019, the United States recorded more than 7500 robbery incidents.^6^ In the United Kingdom, as well, recent reports highlight a continuing trend of pharmacy-related crime, with offenders receiving custodial sentences for burglary incidents,^11^ and others being arrested on suspicion robbery at community pharmacies.^10^

Across the Middle East, frequent media reports describe pharmacy robberies in Iraq,^17^ Jordan,^18^ Lebanon,^19^ and Egypt.^20^ However, to the best of our knowledge, no peer-reviewed studies to date provide reliable incidence or prevalence estimates of robbery acts targeting retail community pharmacies in the region. This conclusion is based on a structured literature search of PubMed, Scopus, and Web of Science (January 2000–June 2025) using combinations of terms such as “community pharmacy”, “retail pharmacy”,” community pharmacy”, “robbery”, “crime”, “violence”, “burglary”, and “Middle East”, which did not identify any studies reporting pharmacy-level crime statistics for the region. Although news reports and qualitative evidence, indicate a notable risk, quantitative data remain limited.^5^

Pharmacy-related threats, despite their clear implications for healthcare and staff safety, remain underexplored dimensions of pharmaceutical practice and pharmacy administration science. This study aimed to investigate pharmacy robbery in Jordanian community pharmacies from the perspective of pharmacists. The specific objectives were to: (1) quantify the self-reported occurrence of exposure to robbery attempts among community pharmacists; (2) assess pharmacists' perceptions of pharmacy-level robbery risk factors that may increase the likelihood of robbery; and (3) compare, between pharmacists exposed and not exposed to robbery attempts, both the proportion endorsing each robbery risk factor and the resulting rank order of these factors.

## 2 Method

To achieve the objectives of this cross-sectional study, we administered an online survey using a validated and reliable questionnaire.

### 2.1 Setting

The survey was administered as an online questionnaire and was accessible between 1 September 2023 and 30 September 2024. Recruitment was undertaken using non-probability convenience sampling. The extended data-collection window was maintained to maximize reach across governorates and work shifts and to achieve a robust overall sample, including an adequate number of pharmacists with self-reported robbery exposure to support comparisons by exposure status and multivariable analysis.

The survey link was disseminated via nationwide professional WhatsApp groups and Facebook pages specialized for community pharmacies across Jordan. This strategy leveraged the widespread use of digital professional communication among pharmacy staff and facilitated participation from individuals working in different governorates and across varied work shifts, irrespective of their geographic location or working hours.

### 2.2 Participants

Inclusion criteria: Pharmacists and pharmacist assistants, Students who had completed the qualification training (1400 training hours at the community pharmacies, final semester pharmacy students).

Exclusion criteria: Cases where two pharmacists worked in the same pharmacy, ensuring that only one response per pharmacy was collected. (Only one response per pharmacy was collected, avoid clustering and over-representing individual workplaces, as we explicitly requested one response per pharmacy in both the invitation message and the questionnaire instructions. To prevent duplicate entries—particularly among robbery-exposed pharmacies—submissions were screened using the respondent phone number (as approved in the ethical protocol), and only one record per pharmacy was considered.

Identification and prioritization of factors were collected without direct personal identifiers in the main questionnaire, and responses were analyzed on a de-identified dataset and reported only in aggregate. For respondents who indicated that their pharmacy had experienced a robbery incident, the final survey page included an optional field to provide a mobile telephone number for verification and potential follow-up. This item was clearly labelled as voluntary, and declining to provide contact details had no effect on survey completion or participation. For robbery-exposed respondents who voluntarily provided a phone number, the process was not fully anonymous at the point of contact collection; however, contact details were stored separately from survey responses and were not linked to the analytic dataset used for factor identification/prioritization analyses.

Any contact information provided was stored in an encrypted file separate from the survey dataset and was accessible only to the principal investigator and one designated research team member, both bound by confidentiality obligations. These details were used exclusively to verify the reported robbery incidents and, where applicable, to facilitate follow-up contact; they will be securely destroyed after completion of follow-up activities in accordance with the approved protocol.

Of the 42 respondents reporting robbery exposure, 33 voluntarily provided contact details and 9 remained anonymous. The designated research team member contacted those who provided a number solely to verify the reported incident(s). Verified cases may be used as a sampling frame for a future qualitative study under a different design, subject to additional ethical approval.

### 2.3 Questionnaire development

The initial pool of questionnaire items was drafted by the research team, informed by the available literature and their experience in community pharmacy practice. To strengthen content validity, a multidisciplinary expert panel of eight experts was convened, including representatives from academia (*n* = 2), social sciences (*n* = 1), security (n = 1), community pharmacies (*n* = 3; one of whom had been exposed to a robbery attempt), and law (n = 1). Through panel discussions, factors relevant to the assessment of robbery risk were identified and prioritized for evaluation from the pharmacists' perspective. To ensure content validity, only items achieving Lawshe's Content Validity Ratio (CVR) above 0.741 with a significance level of *p* < 0.05 were retained for analysis.^21^ A subset of questions was removed from the final instrument, with the intention of being utilized in a separate study focusing on legal and regulatory awareness related to robbery and other forms of pharmacy crimes. A subset of questions was removed from the final instrument because it focused mainly on legal regulations and awareness of rules related to robbery and other pharmacy crimes, which did not align with the core aim of this study. These items are better suited to a separate study specifically designed to assess pharmacists' legal and regulatory awareness rather than their perceptions of robbery risk.

To assess internal reliability, a pilot test of the questionnaire was conducted with 20 community pharmacists. Their responses were used solely for evaluation and instrument refinement and were excluded from the final analytical sample. Internal consistency was confirmed using Cronbach's alpha, with all multi-item scales demonstrating coefficients above 0.70, indicating satisfactory reliability.

The questionnaire was divided into two primary sections. The first part focuses on demographic information such as age, gender, and other relevant variables In the second part of the questionnaire, pharmacists evaluated a series of pharmacy-level characteristics to identify which factors they perceived as increasing robbery risk. The questionnaire covered a range of pharmacy-level robbery risk factors, including location (non-crowded streets, affluent and low-income areas), pharmacist gender, number of staff on duty, pharmacy type (chain vs independent), size and expected income, presence of security cameras, and dispensing of controlled drugs. Additional items assessed beliefs that robberies are more likely in pharmacies that remain open late, are often premeditated and preceded by observation, or can be impulsive and unplanned, and that robbery risk depends primarily on offender-related criminal and personal factors. Finally, respondents were asked whether they had ever been exposed to a robbery incident in a community pharmacy, and whether they had encountered customers displaying dangerous or aggressive behavior outside any robbery incident.

Operational definitions, these operational definitions were first specified in Arabic and then refined and agreed upon by the expert panel comprising, where the low expert was the assigned expert to ensure alignment with Jordanian criminal and pharmacy regulations and community pharmacy practice, while remaining consistent with international descriptions of pharmacy robbery and related terms.

Robbery was defined as the theft or attempted theft of medicines, cash, or other valuables from an open, staffed pharmacy, involving the actual use or explicit threat of force against staff or customers.,^4^.^6^ Robbery exposure was defined as self-reported direct experience (i.e., being present/on duty) of ≥1 robbery meeting the above operational definition; respondents were coded as exposed (yes) or not exposed (no) accordingly, using an ever/never recall window for their community-pharmacy work in Jordan. Burglary was defined as unlawful entry into a closed pharmacy premises outside normal opening hours with the intention of stealing medicines, cash, or other property, without direct confrontation with staff,^4^,^12^,^22^.^23^ In line with international frameworks, workplace violence was understood as any incident in which pharmacy staff are abused, threatened, or assaulted in circumstances related to their work, involving an explicit or implicit challenge to their safety, well-being, or health^2^; physical violence as the use of physical force that has a high likelihood of causing physical, sexual, or psychological harm (e.g. hitting, slapping, pushing, or throwing objects)^1^; and verbal violence as threats, insults, shouting, intimidation, humiliation, or other abusive speech directed towards staff.^1^ Dangerous behavior was defined as actions by a customer that pose an immediate threat to the physical safety of staff, other customers, or property (e.g. explicit threats of violence, brandishing a weapon, severe physical intimidation), other than incidents meeting the operational definition of robbery, while Aggressive behavior was defined as hostile or violent verbal or physical actions by a customer that create a tense, threatening, or unsafe environment (e.g. yelling, verbal threats, aggressive gestures, refusal to follow staff instructions), again excluding incidents classified as robbery,^2^,^5^,^9^,^24^.^25^

### 2.4 Sample size calculation

The required sample size was calculated using the single-proportion formula with a finite population correction, based on an estimated population of 3700 registered community pharmacies in Jordan as of September 2023.^26^ Assuming a conservative prevalence estimate of 50% (*p* = 0.50) to maximize variability, a 99% confidence level (Z = 2.576), and a 5% margin of error (e = 0.05), the initial sample size was calculated as N = Z^2^ P (1-p) / e^2^ = 563. Where N = required sample size, Z = *Z*-score (standard normal value) corresponding to the chosen confidence level, which was 99%, p = assumed prevalence / proportion (here 0.50 for 50%), e = desired margin of error (absolute precision), which was 0.05.

### 2.5 Statistical analysis

Statistical analyses were conducted using SPSS version 26 (SPSS Inc., Chicago, IL, USA). Descriptive statistics were used to describe the data. Categorical variables were presented as frequencies and percentages. Chi-squared test was used to assess the association between statements and participants with significant level < 0.05. Item-level missing data were minimal; analyses were conducted on complete cases for the primary outcome and key covariates, and no data imputation was performed. To compare the strength of association between variables, Cramer's V was calculated, which would help in ranking the variables based on the strength of association. Moreover, it would provide insight into the strength of association, where value of 1 indicates complete association, “0” indicates no association, >0.25 indicates a very strong relationship, >0.15 indicates a strong relationship, >0.1 indicates a moderate relationship, and > 0.05 indicates a weak relationship.^27^

Binary logistic regression was conducted to examine the impact of pharmacy- and pharmacist-related factors on the likelihood of robbery exposure (coded 0 = no previous exposure, 1 = exposed robbery incident). A backward stepwise logistic regression approach was employed, starting from a full model in which all candidate predictors were entered at Step 1, including pharmacy location characteristics, pharmacist gender, number of employees, pharmacy type (chain vs independent), pharmacy size and expected income, presence of security cameras/recording systems, dispensing of controlled drugs, and perception/experience constructs related to robbery risk and customer behavior. By initially modelling all variables simultaneously and then sequentially removing non-significant predictors, this backward stepwise procedure helped to control potential confounding between correlated factors and to identify those variables with independent associations with robbery exposure. Model performance was evaluated using overall model significance, explained variance (Nagelkerke R^2^) and goodness-of-fit indices, and after nine steps the procedure yielded a final parsimonious model in which only a restricted set of predictors remained statistically significant.

## 3 Results

### 3.1 Demographics and robbery attempts

Table 1 presents the demographic characteristics of the participants and their exposure status to robbery attempts. A total of 975 participants were surveyed, among whom 42 (4.3%) were exposed to robbery attempts. Gender-stratified analysis showed that male pharmacists (7.5%) had a significantly higher prevalence of exposure to robbery attempts than female pharmacists (3.2%) (χ^2^ = 8.277, *p* = 0.004, Cramer's V = 0.092). Age was significantly associated with exposure to robbery attempts (χ^2^ = 36.980, *p* < 0.001, Cramer's V = 0.195), with participants older than 30 years reporting the highest prevalence exposure (11.7%) compared to younger groups. Regarding occupation, pharmacists showed a higher percentage of exposure (5.3%) than senior students (0.6%) and pharmacy assistant (χ^2^ = 7.445, *p* = 0.024, Cramer's V = 0.087). Overall, exposure to robbery attempts varied significantly by gender, age, and occupational status. However, when considering effect sizes, age emerged as the strongest and most influential demographic correlate of exposure to robbery attempts (Cramer's V = 0.195), followed by gender (0.092) and occupation (0.087).

**Table 1:** Demographic Characteristics of Participants and Their Experience with Robbery Attempts.

Variable	Category	Have you ever been exposed to a robbery incident in a community pharmacy?		Total	Value	*P* value Chi square	Cramer's V
		No (*n* = 933)	Yes (*n* = 42)				
Gender	Female	697	23	720	8.28	0.004	0.09
		96.8%	3.2%	100.0%			
	Male	236	19	255			
		92.5%	7.5%	100.0%			
Age Group	Less than 25	519	10	529	36.98	<0.001	0.19
		98.1%	1.9%	100.0%			
	Between 25 and 30	226	7	233			
		97.0%	3.0%	100.0%			
	More than 30	188	25	213			
		88.3%	11.7%	100.0%			
Participants occupation	Pharmacists	626	35	661	7.45	0.024	0.09
		94.7%	5.3%	100.0%			
	Senior Student	173	1	174			
		99.4%	0.6%	100.0%			
	Pharmacist Assistant	134	6	140			
		95.7%	4.3%	100.0%			

### 3.2 Participants' perceptions towards factors increasing the prospects of robberies

As shown in Table 2, the highest agreement was observed for the belief that the likelihood of robberies has recently increased (92.1%). Participants also identified several pharmacy-level robbery risk factors, including late-night opening hours (81.2%), single staffing (77.3%), dispensing narcotics (77.2%), and absence of surveillance cameras (72.6%), which were perceived as particularly important contributors to robbery risk. The majority of the participants (69.5%) believe that robberies occur after observing the pharmacy for days, whereas a minority (7.3%) think that robberies occur at the moment without prior planning. While 72.1% and 68.0% of the participants dealt with customers who looked dangerous or who reacted inappropriately, respectively, 4.3% of pharmacists reported having been exposed to at least one robbery incident in their workplace.

**Table 2:** Pharmacy staff perceptions of pharmacy-level robbery risk factors.

Statement	Not sure	No	Yes	Total Responses
Pharmacies located in non-crowded areas are at higher risk of robbery.	158 (16.2%)	227 (23.3%)	590 (60.5%)	975
Pharmacies employing mostly female staff are at higher risk of robbery.	199 (20.4%)	258 (26.5%)	518 (53.1%)	975
Pharmacies with only one staff member are at higher risk of robbery	138 (14.2%)	83 (8.5%)	754 (77.3%)	975
Chain pharmacies are more targeted than independent ones	224 (23.0%)	643 (65.9%)	108 (11.1%)	975
Pharmacies without surveillance cameras are at higher risk of robbery.	117 (12.0%)	150 (15.4%)	708 (72.6%)	975
Large, high-revenue pharmacies are at higher risk of robbery.	254 (26.1%)	349 (35.8%)	372 (38.2%)	975
Small, low-revenue pharmacies are at higher risk of robbery	282 (28.9%)	610 (62.6%)	83 (8.5%)	975
Pharmacies located in affluent areas are at higher risk of robbery	366 (37.5%)	361 (37.0%)	248 (25.4%)	975
Pharmacies in low-income areas are at higher risk of robbery	318 (32.6%)	388 (39.8%)	269 (27.6%)	975
Pharmacies that dispense narcotics are at higher risk of robbery	150 (15.4%)	72 (7.4%)	753 (77.2%)	975
Robberies are more likely in pharmacies that remain open late	119 (12.2%)	64 (6.6%)	792 (81.2%)	975
Robberies are often premeditated and preceded by observation	204 (20.9%)	93 (9.5%)	678 (69.5%)	975
Some robberies are impulsive and occur without prior planning	286 (29.3%)	617 (63.3%)	72 (7.4%)	975
Robbery depends primarily on the criminal and related personal factors	293 (30.1%)	217 (22.3%)	465 (47.7%)	975
Over 50% of robbery suspects are eventually apprehended	519 (53.2%)	152 (15.6%)	304 (31.2%)	975
Have you ever been exposed to a robbery incident in a community pharmacy?	N/A	933 (95.7%)	42 (4.3%)	975
Have you ever encountered a customer you considered dangerous a? (Other than any robbery attempt.)	N/A	272 (27.9%)	703 (72.1%)	975
Have you ever dealt with a customer exhibiting aggressive behavior b? (Other than any robbery attempt.)	N/A	312 (32.0%)	663 (68.0%)	975
Do you think the likelihood of robberies has increased recently?	N/A	77 (7.9%)	898 (92.1%)	975

a Dangerous behavior can be defined as actions by a customer that pose an immediate threat to the physical safety of staff, other customers, or property (e.g., threats of violence, brandishing a weapon, physical intimidation). Other than any robbery attempt.

b Aggressive behavior refers to hostile or violent verbal or physical actions by a customer that create a tense, threatening, or unsafe environment (e.g., yelling, threats, aggressive gestures, refusal to follow instructions). Other than any robbery attempt.

A heightened robbery risk was perceived in pharmacies lacking surveillance cameras (72.6%). Pharmacies located in non-crowded areas were also widely viewed as being at higher robbery risk (60.5%), and 53.1% of respondents considered pharmacies employing predominantly female staff to be more exposed to robbery. Additional factors perceived to increase robbery risk included individual criminal or offender-related factors (47.7%), operating as a large, high-revenue pharmacy (38.2%), and situations in which more than half of robbery suspects are apprehended (31.2%). In contrast, perceived robbery risk was lower for pharmacies located in low-income (27.6%) or affluent (25.4%) areas, chain pharmacies (11.1%), and small, low-revenue pharmacies (8.5%).

The findings in Table 2 indicate that pharmacists very frequently encounter customers displaying dangerous (72.1%) or aggressive (68.0%) behavior, which is particularly noteworthy and warrants further attention. Therefore, we conducted additional gender-stratified analyses to better understand differences in responses between male and female pharmacists. Among those reporting encounters with dangerous customers (72.1%, *n* = 703), 72.5% of male pharmacists and 71.9% of female pharmacists reported such experiences, with no statistically significant difference between genders (*p* = 0.89). In contrast, among those reporting encounters with aggressive customers (68.0% overall), male pharmacists reported a significantly higher prevalence than female pharmacists (78.4% vs. 64.3%, *p* < 0.001).

### 3.3 Descriptive statistics: pharmacists' perceptions of pharmacy-level robbery risk factors

See Table 2 here.

### 3.4 Comparisons of robbery risk perceptions by robbery exposure status

Table 3 presents the Chi-square test results, highlighting differences in robbery risk perceptions between pharmacists exposed and not exposed to robbery attempts.

**Table 3:** Chi-squared test, highlighting the differences in risk perceptions between robbery exposed and robbery non-exposed pharmacy groups.

Statement	Answer	Have you ever been exposed to a robbery incident in a community pharmacy?		Asymptotic Significance (2-sided)
		No (n = 933)	Yes (n = 42)	
Small, low-revenue pharmacies are at higher risk of robbery	Not Sure	273	9	**0.001**
		29.3%	21.4%	
	No	587	23	
		62.9%	54.8%	
	Yes	73	10	
		7.8%	23.8%	
Robberies are more likely in pharmacies that remain open late	Not Sure	115	4	**0.004**
		12.3%	9.5%	
	No	56	8	
		6.0%	19.0%	
	Yes	762	30	
		81.7%	71.4%	
Large, high-revenue pharmacies are at higher risk of robbery	Not Sure	251	3	**0.009**
		26.9%	7.1%	
	No	327	22	
		35.0%	52.4%	
	Yes	355	17	
		38.0%	40.5%	
Have you ever dealt with a customer exhibiting **aggressive** behavior?	No	306	6	**0.012**
		32.8%	14.3%	
	Yes	627	36	
		67.2%	85.7%	
Robberies are **impulsive** and occur without prior planning	Not Sure	274	12	**0.012**
		29.4%	28.6%	
	No	595	22	
		63.8%	52.4%	
	Yes	64	8	
		6.9%	19.0%	
Chain pharmacies are more targeted than independent ones	Not Sure	217	7	**0.024**
		23.3%	16.7%	
	No	618	25	
		66.2%	59.5%	
	Yes	98	10	
		10.5%	23.8%	
Pharmacies with **only one staff** member are at higher risk of robbery	Not Sure	135	3	**0.026**
		14.5%	7.1%	
	No	75	8	
		8.0%	19.0%	
	Yes	723	31	
		77.5%	73.8%	
Pharmacies employing mostly female staff are at higher risk of robbery.	Not Sure	195	4	**0.027**
		20.9%	9.5%	
	No	240	18	
		25.7%	42.9%	
	Yes	498	20	
		53.4%	47.6%	
Robbery depends primarily on **the criminal and related personal factors**	Not Sure	283	10	**0.029**
		30.3%	23.8%	
	No	213	4	
		22.8%	9.5%	
	Yes	437	28	
		46.8%	66.7%	
Pharmacies located in affluent areas are at higher risk of robbery	Not Sure	355	11	**0.041**
		38.0%	26.2%	
	No	338	23	
		36.2%	54.8%	
	Yes	240	8	
		25.7%	19.0%	
Pharmacies located in non-crowded areas are at higher risk of robbery.	Not Sure	156	2	0.093
		16.7%	4.8%	
	No	214	13	
		22.9%	31.0%	
	Yes	563	27	
		60.3%	64.3%	
Pharmacies without surveillance cameras are at higher risk of robbery.	Not Sure	112	5	0.295
		12.0%	11.9%	
	No	140	10	
		15.0%	23.8%	
	Yes	681	27	
		73.0%	64.3%	
Pharmacies in low-income areas are at higher risk of robbery	Not Sure	308	10	0.145
		33.0%	23.8%	
	No	373	15	
		40.0%	35.7%	
	Yes	252	17	
		27.0%	40.5%	
Pharmacies that dispense narcotics are at higher risk of robbery	Not Sure	146	4	0.332
		15.6%	9.5%	
	No	67	5	
		7.2%	11.9%	
	Yes	720	33	
		77.2%	78.6%	
Robberies are often premeditated and preceded by observation	Not Sure	195	9	0.997
		20.9%	21.4%	
	No	89	4	
		9.5%	9.5%	
	Yes	649	29	
		69.6%	69.0%	
Over 50% of robbery suspects are eventually apprehended	Not Sure	501	18	0.387
		53.7%	42.9%	
	No	144	8	
		15.4%	19.0%	
	Yes	288	16	
		30.9%	38.1%	
Have you ever encountered a customer you considered **dangerous**?	No	266	6	0.044
		28.5%	14.3%	
	Yes	667	36	
		71.5%	85.7%	
Do you think the likelihood of robberies has increased recently?	No	72	5	0.325
		7.7%	11.9%	
	Yes	861	37	
		92.3%	88.1%	

#### 3.4.1 Gaps differences in perception between the groups

Although a substantial proportion of participants perceived that working late at night increases robbery risk (81.7%), this belief was less prevalent among those exposed to robbery (71.4%). Conversely, while only 6% of all participants believed that late-night operation does not elevate robbery risk, a markedly higher proportion (19%) of pharmacists exposed to robbery reported that remaining open late was not a major robbery risk factor.

Similarly, perceptions regarding large, high-revenue pharmacies differed notably between the overall sample and those exposed to robbery. Nearly half of pharmacists exposed to robbery indicated that large, high-revenue pharmacies are not at increased robbery risk, compared with only 35% of participants in the overall sample who expressed this view, a difference that was statistically significant (*p* = 0.009).

Regarding the impulsiveness of robbery incidents, 6.9% of participants overall characterised robberies as impulsive, whereas 19% of pharmacists **exposed to robbery** reported that the incident they experienced was impulsive (*p* = 0.012). In the same context, while 66.7% of robbery-exposed pharmacists attributed the crime primarily to the personal characteristics of the offender, only 46.8% of participants overall endorsed this view, representing a statistically significant discrepancy (*p* = 0.029).

In relation to pharmacy type, 23.8% of pharmacists exposed to robbery perceived chain pharmacies as being at higher robbery risk, compared with 10.5% of participants in the overall sample, indicating a significant divergence between overall perceptions and the views of those with direct robbery experience (*p* = 0.024).

Finally, the association between staffing and robbery risk was examined. Among pharmacists exposed to robbery, 19% identified having only one staff member on duty as a robbery risk factor, whereas only 8% of participants in the overall sample shared this perception (*p* = 0.026), indicating a marked divergence in how single staffing is perceived in relation to robbery risk by those with and without exposure to robbery.

#### 3.4.2 Similarities in perceptions between exposure groups

Several factors showed no statistically significant differences between the perceptions of the overall sample and the views of pharmacists exposed to robbery incidents. The belief that dispensing narcotics is a robbery risk factor was consistently endorsed across both groups (*p* = 0.332). Perceptions that pharmacies located in non-crowded areas are at higher robbery risk were also comparable between groups (*p* = 0.093). Similarly, no significant differences were observed regarding the belief that pharmacies without surveillance cameras are at higher robbery risk (*p* = 0.295) or that pharmacies located in low-income areas are more at risk of robbery (*p* = 0.145). In addition, the view that robberies are often premeditated and preceded by prior observation showed almost identical levels of agreement between participants overall and those exposed to robbery (*p* = 0.997). Other non-significant differences included the belief that more than 50% of robbery suspects are eventually apprehended (*p* = 0.387) and the perception that the likelihood of robberies has increased recently (*p* = 0.325). Taken together, these findings indicate that, for several key robbery risk factors, participants' perceptions were broadly aligned with the experiences of pharmacists exposed to robbery.

### 3.5 Comparing ranks for variables based on the experience of being robbed

Table 4 presents the comparative ranking of pharmacy-level robbery risk factors among pharmacists exposed and not exposed to robbery attempts, based on the proportion in each group who agreed that each statement represented a robbery risk factor. In both groups, the highest-ranked item was the perception that the likelihood of robberies has increased recently (88.1% among pharmacists exposed to robbery vs 92.3% among those not exposed; no rank difference). Among pharmacists exposed to robbery, the next most prominent items were having dealt with an aggressive customer and having encountered a customer considered dangerous (both 85.7%), followed by the belief that pharmacies dispensing narcotics are at higher risk of robbery (78.6%) and that pharmacies with only one staff member are at higher risk (73.8%). In contrast, among pharmacists not exposed to robbery, late opening ranked second (81.7%), followed by single staffing (77.5%) and dispensing narcotics (77.2%), indicating that extended opening hours and single staffing are particularly salient perceived risk factors in the non-exposed group.

**Table 4:** The comparative ranking analysis among pharmacists exposed and not exposed to robbery attempts.

Rank – exposed to robbery	Robbery risk factor statement	% “Yes” exposed to robbery	% “Yes” not exposed to robbery	Rank – not exposed to robbery	Rank difference^a^	Statistically significant^b^
1	Do you think the likelihood of robberies has increased recently?	88.1	92.3	1	0	No
2	Have you ever encountered a customer you considered dangerous?	85.7	71.5	6	+4	Yes
2	Have you ever dealt with a customer exhibiting aggressive behavior?	85.7	67.2	8	+6	Yes
3	Pharmacies that dispense **narcotics** are at higher risk of robbery	78.6	77.2	4	+1	No
4	Pharmacies with only **one staff member** are at higher risk of robbery	73.8	77.5	3	−1	Yes
5	Robberies are more likely in pharmacies that remain open late	71.4	81.7	2	−3	Yes
6	Robberies are often premeditated and preceded by observation	69	69.5	7	+1	No
7	Robbery depends primarily on the criminal and related personal factors	66.7	46.8	11	+4	Yes
8	Pharmacies without surveillance cameras are at higher risk of robbery.	64.3	73	5	−3	No
8	Pharmacies located in non-crowded areas are at higher risk of robbery.	64.3	60.3	9	+1	No
9	Pharmacies employing mostly female staff are at higher risk of robbery.	47.6	53.4	10	+1	Yes
10	Large, high-revenue pharmacies are at higher risk of robbery	40.5	38	12	+2	Yes
10	Pharmacies in low-income areas are at higher risk of robbery	40.5	27	14	+4	No
11	Over 50% of robbery suspects are eventually apprehended	38.1	30.8	13	+2	Yes
12	Chain pharmacies are more targeted than independent ones	23.8	10.5	16	+4	Yes
12	Small, low-revenue pharmacies are at higher risk of robbery	23.8	7.8	17	+5	Yes
13	Pharmacies located in affluent areas are at higher risk of robbery	19	25.7	15	+2	Yes
13	Robberies are impulsive and occur without prior planning	19	6.8	18	+5	Yes

a Rank difference, Positive rank difference = factor ranked as more important among pharmacists exposed to robbery. Negative rank difference = factor ranked as less important among pharmacists exposed to robbery.

b Based on chi-square tests reported in Table 3.

The rank-difference analysis in Table 5 shows which robbery risk factors became more or less salient following robbery exposure. Positive rank differences (higher importance among pharmacists exposed to robbery) were observed for several items, including having encountered a dangerous customer (+4 ranks), having dealt with an aggressive customer (+6), the belief that robbery risk depends primarily on the offender and related personal factors (+4), chain pharmacies being more targeted than independent ones (+4), pharmacies in low-income areas (+4), small low-revenue pharmacies (+5), and the view that some robberies are impulsive and occur without prior planning (+5). Conversely, negative rank differences indicated reduced perceived importance among robbery-exposed pharmacists for remaining open late (−3 ranks) and the absence of surveillance cameras (−3). Several other robbery risk factors showed little or no shift in rank between groups—such as increased likelihood of robberies overall (0), dispensing narcotics (+1), premeditation and prior observation (+1), non-crowded locations (+1), predominantly female staff (+1), and pharmacies in affluent areas (+1)—suggesting a broadly shared baseline recognition of these risks regardless of direct robbery exposure.

**Table 5:** Multivariable logistic regression of exposure to robbery.

Risk factors for exposure to robbery versus no exposure	Odds Ratio (OR)	95% C.I.for OR		Significance
		Lower	Upper	
Chain pharmacies are more targeted than independent ones				0.037
No	0.339	0.116	0.992	0.048
Yes	0.318	0.128	0.787	0.013
Large, high-revenue pharmacies are at higher risk of robbery				0.033
No	0.188	0.052	0.685	0.011
Yes	0.942	0.454	1.953	0.873
Small, low-revenue pharmacies are at higher risk of robbery				0.001
No	0.290	0.113	0.743	0.010
Yes	0.218	0.100	0.476	0.000
Pharmacies located in affluent areas are at higher risk of robbery				0.028
No	1.270	0.503	3.207	0.613
Yes	0.250	0.100	0.627	0.003
Robberies are more likely in pharmacies that remain open late				0.060
No	0.714	0.322	1.584	0.408
Yes	0.267	0.088	0.806	0.019
Robberies are impulsive and occur without prior planning				0.051
No	0.437	0.165	1.159	0.096
Yes	0.328	0.134	0.804	0.015
Robbery depends primarily on the criminal and related personal factors				0.001
No	**1.266**	0.403	3.973	0.687
Yes	**6.282**	2.482	15.904	0.000
Have you ever dealt with a customer exhibiting aggressive behavior? (Yes)	3.108	1.246	7.755	0.015

### 3.6 Regression, multivariable model

Table 5 presents the multivariable binary logistic regression analysis used to identify statistically significant risk factors for exposure to robbery versus no exposure, adjusting for all other variables in the model and applying a backward stepwise selection approach to reduce potential confounding. In the final Step-9 model, the retained correlates of robbery exposure were restricted to a focused set of constructs: pharmacy type (chain vs independent), pharmacy size and economic profile (large high-revenue and small low-revenue outlets), pharmacy location in affluent areas, opening-hours profile (late-opening pharmacies), attributions about offender behavior (impulsivity and offender-related personal factors), and prior exposure to aggressive customers. For most of these constructs the overall Wald test indicated a statistically significant association with robbery exposure; for two perception items—“Robberies are more likely in pharmacies that remain open late” (overall *p* = 0.060) and “Robberies are impulsive and occur without prior planning” (overall *p* = 0.051) the overall effects were attenuated to borderline non-significance, but they were retained in the model because at least one response category within each remained significantly associated with robbery exposure (p < 0.05). In contrast, other factors entered at Step 1—pharmacy location characteristics (crowded vs non-crowded and more vs less affluent areas), staff gender composition, number of staff on duty, security technology (presence of surveillance cameras/recording systems), dispensing of controlled drugs, beliefs about premeditated/observed robberies, and prior encounters with “dangerous” customers—did not remain statistically significant after adjustment and were therefore not independently associated with robbery exposure in the final model.

The final multivariable logistic regression model showed excellent performance, with a very high Nagelkerke R^2^ of 0.860, indicating that the predictors explained a large proportion of the variance in robbery exposure and provided strong discrimination between exposed and non-exposed pharmacists. Although the Hosmer–Lemeshow test suggested some deviation from perfect calibration (χ^2^ = 17.61, df = 8, p = 0.024), the overall model fit remained statistically robust (Omnibus χ^2^ = 1008.94, df = 14, p < 0.001).

In the final multivariable model, the strongest risk gradient was observed for the belief that robbery risk depends primarily on the offender and related personal factors: pharmacists who agreed with this statement had markedly higher odds of exposure to robbery (OR = 6.28, 95% CI 2.48–15.90; p < 0.001), whereas disagreement was not significantly associated with exposure (OR = 1.27, 95% CI 0.40–3.97; *p* = 0.687). The next strongest risk marker was prior exposure to aggressive customers, with pharmacists who had ever dealt with an aggressive customer showing significantly higher odds of robbery exposure (OR = 3.11, 95% CI 1.25–7.76; *p* = 0.015). Several perceptions were instead associated with lower odds of exposure. For chain versus independent pharmacies, both agreement and disagreement that chain pharmacies are more targeted were linked to similarly reduced odds of robbery exposure (Yes: OR = 0.32, 95% CI 0.13–0.79; *p* = 0.013; No: OR = 0.34, 95% CI 0.12–0.99; *p* = 0.048). For small, low-revenue pharmacies, both agreement and disagreement that these pharmacies are at higher risk were also protective, with slightly lower odds among those who agreed (Yes: OR = 0.22, 95% CI 0.10–0.48; *p* < 0.001; No: OR = 0.29, 95% CI 0.11–0.74; *p* = 0.010). Disagreement that large, high-revenue pharmacies are at higher risk was associated with substantially reduced odds (OR = 0.19, 95% CI 0.05–0.69; *p* = 0.011), whereas agreement was not significant (OR = 0.94, 95% CI 0.45–1.95; *p* = 0.873). Agreement that pharmacies in affluent areas are at higher risk (OR = 0.25, 95% CI 0.10–0.63; *p* = 0.003), that robberies are more likely in pharmacies that remain open late (OR = 0.27, 95% CI 0.09–0.81; *p* = 0.019), and that some robberies are impulsive and occur without prior planning (OR = 0.33, 95% CI 0.13–0.80; *p* = 0.015) was likewise associated with lower odds of robbery exposure, while the corresponding “No” categories for these items did not reach statistical significance.

## 4 Discussion

This cross-sectional study provides the first large, nationwide sample evidence on pharmacy robberies in Jordanian community pharmacies and directly addresses our stated objectives. Robbery emerged as an infrequent but tangible risk (4.3% reported at least one robbery attempt), occurring against a background of widespread exposure to dangerous (72.1%) and aggressive (68.0%) customer behavior. Pharmacy staff most frequently identified late-night opening, single staffing, dispensing of narcotics, and absence of surveillance cameras as pharmacy-level characteristics that increase robbery risk, and these perceptions, together with systematic differences in rankings between robbery-exposed and non-exposed staff, were further clarified through multivariable modelling of independent correlates of robbery exposure.

In total, 975 responses were analyzed, with each respondent representing a different community pharmacy. This pharmacy-level sample exceeds those of recent studies on workplace violence among pharmacists in Saudi Arabia (*n* = 319)^2^ and Southeast Europe (*n* = 732),^1^ which examined violence more broadly rather than robbery specifically, and is also larger than the US study of pharmacy students on curricular addition of robbery education (*n* = 285), which focused on educational needs rather than robbery risk in practice settings.^6^

In our survey, 4.3% of respondents reported that their community pharmacy had experienced robbery incident, indicating that robbery is an infrequent but tangible event in Jordanian community pharmacies. This prevalence is lower than the lifetime robbery exposure reported among community pharmacy staff in Southeast Europe (26%).^1^ Nevertheless, robbery is clearly recognized as a significant risk in community pharmacy practice and as a very traumatic event for those who experience it.^1^ This is further evidenced by recent calls for dedicated robbery-related content in pharmacy curricula in the United States.^6^

Beyond exposure to robbery incidents, our data indicates that 72.1% of respondents had encountered customers displaying dangerous behavior and 68.0% had encountered aggressive behavior. These proportions are consistent with the broader literature on workplace violence against pharmacists, including a recent meta-analysis estimating that around 45% of pharmacists have experienced workplace violence, with 50% reporting verbal abuse, 42% threats and 27% physical assaults^3^ and with cross-sectional studies from Southeast Europe, where more than 80% of community pharmacy staff reported verbal violence at work,^1^ in Saudi Arabia approximately half of pharmacists reported at least one form of workplace violence, predominantly verbal.^2^ In our study, 92.1% of participants believed that the likelihood of robbery had increased, underscoring a pervasive sense of vulnerability among pharmacy staff and the reported perceived potential for everyday aggression escalation into more serious violent events,^2^.^15^

In the present study, **male** staff reported higher robbery exposure than females (7.5% vs. 3.2%), and those **aged** > 30 years were more likely to have experienced a robbery than those <25 years (11.7% vs. 1.9%). These gradients indicate that robbery risk is concentrated among more experienced staff and are consistent with previous reports from Shelley and the Pharmacists Mutual Insurance Company that male gender, longer tenure, increase vulnerability to pharmacy crime,^22^.^25^ Moreover, in both robbery-exposed and non-exposed groups, dispensing narcotics, late-night opening and single staffing were consistently ranked as key robbery risk factors. This perception is consistent with external evidence that solo workers, particularly in retail and late-night settings, face elevated risks of robbery and violence, and that offenders frequently prefer targets with few or lone employees,^1^,^22^.^25^

In the present study, the final backward stepwise logistic regression, the model showed high explanatory power (Nagelkerke R^2^ = 0.860). The strongest independent correlate of robbery exposure was the belief that robbery risk depends primarily on offender-related personal factors (OR = 6.28, 95% CI 2.48–15.90; p < 0.001), followed by prior experience of aggressive customers (OR = 3.11, 95% CI 1.25–7.76; p = 0.015). These findings are consistent with offender-focused accounts of pharmacy crime and wider workplace-violence evidence that emphasise offender motivation, drug-seeking and escalating aggression as key drivers of serious incidents,^3^,^22^.^25^

In contrast, factors often treated as “classic” environmental risk markers, such as pharmacy type, economic profile, affluent location, late opening hours, and single-staffing,^3^,^22^ showed – in our multivariable regression model- weaker associations with reported exposure after adjustment of other variables. Moreover, hardware-focused features (e.g., lack of cameras) were not independently associated with exposure.

Although the broader literature on surveillance in public and occupational settings remains mixed and continues to argue that cameras serve both deterrent and investigative functions,^28^, ^29^, ^30^ our findings—across both the risk-ranking results and the regression model—support that surveillance cameras at pharmacies may operate for investigative (detective) measure, rather than as an independently protective or deterrent factor against robbery exposure,^31^.^32^ This interpretation is aligned with offender-interview evidence indicating that internal cameras were commonly viewed as unimportant for target selection, largely because offenders assumed cameras are ubiquitous, can be mitigated through disguises, or are poorly positioned or nonfunctional.^22^

The results support the view that offender- and behavior centered cues—particularly repeated aggressive customer encounters—may be more informative for understanding robbery risk than structural premises characteristics alone. This aligns with evidence from the broader criminological concept of repeat victimization, where some offenders return to the same target when conditions remain unchanged (Weisel 2005), suggesting that prior incidents and recurrent offender–pharmacy interactions can be more indicative of subsequent risk than static premises features,^22^,^33^.^34^

### 4.1 Limitations

This study has a few key limitations that should be acknowledged. First, the cross-sectional survey design captures perceptions and experiences at a single point in time and therefore cannot account for temporal changes in crime patterns or pharmacy operations. Second, reliance on self-reported data introduces the potential for recall and social desirability bias, which may affect the accuracy of reported robbery exposure and robbery risk perceptions. Third, the use of an online questionnaire disseminated via social media platforms may have introduced selection bias, as participants with stronger opinions or prior exposure to crime might have been more inclined to respond. In addition, only one response per pharmacy was collected to avoid clustering and over-representing individual workplaces; as a result, within-pharmacy variation in staff perceptions could not be systematically examined. Nonetheless, dissemination via nationwide professional WhatsApp groups and Facebook pages, combined with a relatively large and heterogeneous sample of pharmacists, likely enhanced respondent diversity and statistical precision, partially mitigating concerns about selection bias—particularly for perceptions of pharmacy-level robbery risk factors. Although the regression model exhibited high explanatory power (Nagelkerke R^2^ = 0.860), the limited number of robbery events means that overfitting cannot be excluded, and the estimates should be interpreted with caution. Robbery exposure was assessed as a binary self-report (ever vs never); the questionnaire did not capture the number of incidents (single vs multiple exposures) nor the timing, recency of the exposure. Finally, because the study was conducted exclusively in Jordan, the findings may not be directly generalizable to settings with different regulatory, social, or economic contexts, and the absence of longitudinal data or qualitative follow-up limits the ability to explore how robbery risk perceptions and responses evolve over time or are shaped by local context.

### 4.2 Strengths

Despite these limitations, the study has several important strengths. It represents, to our knowledge, the first academic investigation of pharmacy robberies in Jordan, addressing a previously unexplored area of pharmacy safety research. The survey instrument underwent structured content validation by a multidisciplinary panel, ensuring that the included robbery risk factors and exposure items were both contextually relevant and evidence-informed. The large and diverse sample of 975 participants provides broad coverage of the community pharmacy workforce and enhances the stability and internal validity of the estimated associations. The explicit comparison between pharmacists exposed and not exposed to robbery, in terms of both reported experiences and perceived pharmacy-level robbery risk factors, offers novel insights into perception–experience gaps that are directly relevant to risk assessment and management in practice. Furthermore, by situating the Jordanian findings alongside emerging international evidence on pharmacy violence and robbery^1^–,^3^,^6^ this study contributes context-specific data to the wider global discourse on community pharmacy safety and security.

## 5 Conclusion

This study provides the first systematic evidence on pharmacy robbery in Jordanian community pharmacies, showing that robbery occurs within a broader context of highly prevalent dangerous and aggressive customer behavior. Pharmacists consistently identified late opening, single staffing, dispensing narcotics and lack of surveillance cameras as key pharmacy-level robbery risk factors, and several of these factors were appraised differently by pharmacists exposed and not exposed to robbery, suggesting that direct exposure to robbery shapes how risk is perceived.

Offender- and behavior-focused factors—particularly beliefs about offender motivation and prior aggressive customer encounters—were more strongly associated with robbery exposure than traditional environmental markers such as pharmacy type, location or security hardware. These findings support the need for evidence-informed security policies in community pharmacies, including minimum staffing during high-risk periods, appropriate surveillance and alarm systems, and targeted training to recognize and manage high-risk customer behavior, alongside improved crime reporting and further longitudinal and qualitative research.

## 6 Future recommendations

From a practical perspective, pharmacy owners and policymakers should prioritise comprehensive security protocols in community pharmacies, and regulations requiring a minimum of two staff members during identified high-risk periods (such as late opening hours). These measures should be complemented by structured training in risk recognition and conflict de-escalation, with a particular focus on managing dangerous and aggressive customer behavior. Regulatory bodies could also mandate standardised reporting systems for pharmacy crime to improve data accuracy and enable monitoring of robbery trends over time.

For future research, longitudinal studies are needed to frameworks influence robbery risk over time in community pharmacies. In addition, qualitative or mixed-methods studies exploring offender motivations and decision-making, alongside the perspectives of pharmacists exposed and not exposed to robbery, would complement the present findings and help to inform more targeted, behavior-centred prevention strategies.

## Declaration of generative AI and AI-assisted technologies in the manuscript preparation process

During the preparation of this work, the authors used *ChatGPT (OpenAI)* as a language-support tool to assist with translating (Arabic – English), editing, clarifying wording, and improving the coherence and academic style of the manuscript. No AI tools were used for data collection, data analysis, or the generation or interpretation of study results. After using this tool, the authors thoroughly reviewed, revised, and edited all content and took full responsibility for the integrity and accuracy of the final manuscript.

## CRediT authorship contribution statement

**Mohanad Odeh:** Writing – review & editing, Validation, Supervision, Project administration, Methodology, Investigation, Formal analysis, Data curation, Conceptualization. **Dana M. Odeh:** Writing – review & editing, Methodology, Formal analysis, Conceptualization. **Enas Alkhader:** Writing – review & editing, Software, Resources, Formal analysis, Data curation. **Duaa Alzyoud:** Writing – original draft, Visualization, Software.

## Declaration of competing interest

The authors received no financial support for this research and declare no conflicts of interest.

## References

[1] Popčević M., Javorina T., Košiček M., Meštrović A. (2024). Exposure of pharmacists and pharmacy technicians to violence in community pharmacies in southeast europe: frequency and ethical considerations. Pharm (Basel, Switzerland).

[2] Alhomoud F. (2025). Violence in the workplace towards pharmacists working in different settings in saudi arabia: a cross-sectional study. Safety.

[3] Bhagavathula A.S., Obamiro K., Hussain Z., Tesfaye W. (2023). Workplace violence against pharmacists: a systematic review and meta-analysis. J Am Pharm Assoc.

[4] La Vigne N., Wartell J. (2015). https://www.govinfo.gov/app/details/GOVPUB-J36-PURL-gpo92070.

[5] Khazen M., Guttman N. (2022). Pharmacists under pressure to concede: why pharmacists provide non-prescribed antibiotics in the Arab minority in Israel. SSM - Qual Res Heal.

[6] McFarland A., Devine P.S. (2024). Curricular addition of pharmacy robbery education. Curr Pharm Teach Learn.

[7] Dugato M., Sidoti C. (2025). The organised theft of medicines: a study of the methods for stealing and reselling medicines and medical devices in the EU and beyond. Eur J Crim Policy Res.

[8] Yagil D. (2017). There is no dark side of customer aggression − It’s all dark. J Mark Manag.

[9] Irish Pharmacy Union, Crimes Experienced by Pharmacies Continue to Rise, Irish Pharmacy Union, Ireland, 2025. https://ipu.ie/communication/crimes-experienced-by-pharmacies-continue-to-rise/#:∼:text=The IPU Crime Survey 2025,experiencing 10 or more incidents.

[10] Neil Trainis (2024). Man Arrested on Suspicion of Robbery at Boots and Kamsons Pharmacies, Pharmacy Magazine, Sussex. https://www.pharmacymagazine.co.uk/people-news/man-arrested-on-suspicion-of-robbery-at-boots-and-kamsons-pharmacies.

[11] Neil Trainis (2024). https://www.pharmacymagazine.co.uk/news/two-men-handed-six-month-prison-sentences-for-burglary-at-mansfield-pharmacy.

[12] Neil Trainis (2025). https://www.pharmacymagazine.co.uk/news/pharmacy-staff-wear-panic-button-devices-like-necklaces-to-stop-criminals.

[13] Neil Trainis (2025). Man Caused ‘Fear or Alarm’ After Shouting and Swearing at Pharmacy Staff, Pharmacy Magazine, Scotland. https://www.pharmacymagazine.co.uk/news/man-caused-fear-or-alarm-after-shouting-and-swearing-at-pharmacy-staff.

[14] Burghle A. (2020). Customers’ information seeking behavior prior to community pharmacy visits: a community pharmacy survey. Res Soc Adm Pharm.

[15] Yasmeen A. (2023). Suspected inappropriate use of prescription and non-prescription drugs among requesting customers: a Saudi community pharmacists’ perspective. Saudi Pharm J.

[16] Odeh M., Tailakh H.M., Al Bawab A.Q.F., Elsahoryi N.A., Alzoubi K.H. (2022). A comprehensive assessment of knowledge, attitudes, and practicalities related to doping agents use among jordanians. Clin Pract Epidemiol Ment Health.

[17] Alsumaria (2025). Arabic Title: السطو على الصيدليات - خط احمر [Pharmacy Robberies: A Red Line] *News Paper Report*, Iraq. https://www.youtube.com/watch?v=qN17NzwVTpQ.

[18] Garaanews (2025). Arabic Title: عصابة السطو على الصيدليات في قبضة الامن [Pharmacy Robbery Gang Arrested by Security Forces]., News Paper Report, Amman. https://garaanews.com/article/187423.

[19] Sawt Beirut International (2025). Arabic Title: توقيف منفّذ عمليات السطو على الصيدليات [Detention of the Person Who Carried Out Pharmacy Robbery Operations]., News Paper Report, Beirut. https://www.sawtbeirut.com/lebanon-news/توقيف-منفّذ-عمليات-السطو-على-الصيدليا/.

[20] Elwatan News (2025). Arabic Title: ضبط 3 أشخاص بتهمة سرقة 600 ألف جنيه من صيدلية في الجيزة [Arrest of Three Suspects Accused of Stealing EGP 600,000 From a Giza Pharmacy]., News Paper Report, Cairo - Jeeza. https://www.elwatannews.com/news/details/7834325.

[21] Wilson F.R., Pan W., Schumsky D.A. (2012). Recalculation of the critical values for Lawshe’s content validity ratio. Meas Eval Couns Dev.

[22] Shelley T.O.C. (2016). Pharmacy robbery and burglary : the offender perspective. Cent Study Crime Just Dep Sociol Color State Univ.

[23] Ekwall D., Brüls H., Wyer D. (2016). Theft of pharmaceuticals during transport in Europe. J Transp Secur.

[24] Steele R. (2024). Crime, poverty and survival in the Middle East and North Africa: the ‘dangerous classes’ since 1800. Middle East Stud.

[25] Pharmacists Mutual Insurance Company (2015). Pharmacy Crime A Review of Pharmacists Mutual Experience. https://naspa.us/wp-content/uploads/2016/06/PMC-Crime-Report.pdf.

[26] Jordanian Pharmacist Association (2023). Pharmacies in Jordan, The Pharmacies Market in Jordan. https://www.jpa.org.jo/.

[27] Akoglu H. (2018). User’s guide to correlation coefficients. Turkish J Emerg Med.

[28] Piza E.L., Welsh B.C., Farrington D.P., Thomas A.L. (2019). CCTV surveillance for crime prevention. A 40-year systematic review with meta-analysis. CrimRxiv.

[29] Piza E.L. (2018). The crime prevention effect of CCTV in public places: a propensity score analysis. J Crime Justice.

[30] Robin L., Peterson B.E., Lawrence D.S. (2021). How do close-circuit television cameras impact crimes and clearances? An evaluation of the Milwaukee police department’s public surveillance system. Police Pract Res.

[31] Thomas A.L., Piza E.L., Welsh B.C., Farrington D.P. (2022). The internationalisation of cctv surveillance: Effects on crime and implications for emerging technologies. Int J Comp Appl Crim Just.

[32] Matczak P., Wójtowicz A., Dąbrowski A., Leitner M., Sypion-Dutkowska N. (2023). Effectiveness of CCTV systems as a crime preventive tool: evidence from eight Polish cities. Int J Comp Appl Crim Just.

[33] Raaijmakers N. (2023). Assessing the predictive validity of a risk assessment instrument for repeat victimization in the Netherlands using prior police contacts. Eur J Criminol.

[34] Estévez-Soto P.R., Johnson S.D., Tilley N. (2021). Are repeatedly extorted businesses different? A multilevel hurdle model of extortion victimization. J Quant Criminol.

